# S2k guideline for diving accidents

**DOI:** 10.3205/000315

**Published:** 2023-03-03

**Authors:** Björn Jüttner, Christian Wölfel, Claudio Camponovo, Holger Schöppenthau, Johannes Meyne, Carmen Wohlrab, Henning Werr, Till Klein, Giso Schmeißer, Karsten Theiß, Philipp Wolf, Oliver Müller, Thorsten Janisch, Johannes Naser, Susanne Blödt, Cathleen Muche-Borowski

**Affiliations:** 1 German Diving and Hyperbaric Medical Society (GTÜM); 2 Swiss Underwater and Hyperbaric Medical Society (SUHMS); 3 German Interdisciplinary Association for Intensive Care and Emergency Medicine (DIVI); 4 German Recreational Divers Association (VDST); 5 Naval Medical Institute of the German Navy (SchiffMedInstM); 6 Association of German Hyperbaric Treatment Centers (VDD); 7 German Society for Occupational and Environmental Medicine (DGAUM); 8 German Life-Saving Society (DLRG); 9 German Red Cross (DRK), Water Rescue Service; 10 German Society of Anaesthesiology and Intensive Care Medicine (DGAI); 11 Professional Association of German Anaesthesiologists (BDA); 12 Association of the Scientific Medical Societies in Germany (AWMF)

**Keywords:** diving accident, decompression sickness, decompression illness, arterial gas embolism, oxygen, hyperbaric oxygen therapy

## Abstract

For the purposes of this guideline, a diving accident is defined as an event that is either potentially life-threatening or hazardous to health as a result of a reduction in ambient pressure while diving or in other hyperbaric atmospheres with and without diving equipment.

This national consensus-based guideline (development grade S2k) presents the current state of knowledge and recommendations on the diagnosis and treatment of diving accident victims. The treatment of a breath-hold diver as well as children and adolescents does not differ in principle.

In this regard only unusual tiredness and itching without visible skin changes are mild symptoms.

The key action statements: on-site 100% oxygen first aid treatment, immobilization/no unnecessary movement, fluid administration and telephone consultation with a diving medicine specialist are recommended.

Hyperbaric oxygen therapy (HBOT) remains unchanged as the established treatment in severe cases, as there are no therapeutic alternatives. The basic treatment scheme recommended for diving accidents is hyperbaric oxygenation at 280 kPa.

## Abstract (de)

Ein Tauchunfall im Sinne dieser Leitlinie ist ein potenziell lebensbedrohliches oder gesundheitsschädigendes Ereignis, hervorgerufen durch Abfall des Umgebungsdruckes beim Tauchen oder aus sonstiger hyperbarer Atmosphäre mit und ohne Tauchgerät.

Diese nationale S2k-Leitlinie legt den aktuellen Stand der Erkenntnisse und der konsentierten Empfehlungen in der Diagnostik und Behandlung von Patienten nach Tauchunfällen dar. Die Behandlung von Apnoetauchern sowie Kindern und Jugendlichen unterscheidet sich prinzipiell nicht.

Milde Symptome sind nur die auffällige Müdigkeit und ein Hautjucken ohne sichtbare Hautveränderungen.

Wesentliche Bedeutung bei der Versorgung von Tauchunfällen hat die frühzeitige Atmung von 100%igem Sauerstoff. Weiterhin werden die Ruhiglagerung/keine unnötige Bewegung, eine moderate Flüssigkeitsgabe und eine Taucherärztliche Telefonberatung empfohlen.

Die hyperbare Sauerstofftherapie (HBOT) ist bei schweren Dekompressionsunfällen unverändert ohne therapeutische Alternative. Als Behandlungsschema wird grundsätzlich eine HBOT bei 280 kPa empfohlen.

## 1 Introduction

### 1.1 Objective

This guideline represents the current state of knowledge and recommendations on the diagnosis and treatment of diving accident victims with regard to:

- First aid by lay persons as well as treatment by medical assistants and physicians
- The sequence of rescue chain deployment and the transportation of diving accident victims
- Initial hyperbaric medical treatment of diving accident victims
- The further medical care of diving accident victims

### 1.2 Basic methodological principles

The methodological approach adopted in the development of the guideline is set out in the Guideline Report. This is freely available online, e.g., on the AWMF website (http://www.awmf.org/).

#### 1.2.1 Definitions used for strengths of recommendation and consensus

##### 1.2.1.1 Formulation of the strength of recommendations

- Strong recommendation: shall/shall not
- Recommendation: should/should not
- Open recommendation: can/can be dispensed with

##### 1.2.1.2 Classification of strength of consensus

- Strong consensus: agreement between >95% of participants
- Consensus: agreement between >75–95% of participants
- Majority agreement: agreement between >50–75% of participants
- No consensus: agreement between <50% of participants

#### 1.2.2 Period of validity and update procedure

This S2k guideline is valid until November 30, 2027. Regular updates are foreseen. If amendments are urgently required, these will be published separately. Comments and suggestions for the updating process are expressly desired and can be sent to the following address:

Gesellschaft für Tauch- und Überdruckmedizin (GTÜM e.V.), Professor-Küntscher-Straße 8, 82418 Murnau am Staffelsee, Germany, gtuem@gtuem.org

## 2 Definition, pathophysiology, and prevention

### 2.1 Definition

What is the definition of a “diving accident”?

*For the purposes of this guideline, a “diving accident” is defined as an event that is either potentially life-threatening or hazardous to health as a result of a reduction in ambient pressure while diving or in other hyperbaric atmospheres with and without diving equipment.*

– Yes: 11/11, no: 0, abstentions: 0

– Strength of consensus: 100% (strong consensus)

The suspected diagnosis of “diving accident” is likely in the presence of the following conditions [1]:

- breathing was performed using diving equipment under water, irrespective of the breathing gas/breathing gas mixture used (potentially only a single breath),

or

- breathing was performed using air that had collected under water (e.g., in a wreck or cave),

or

- breath-hold dives were performed (generally several deep dives) [2], [3] and
- mild and/or severe symptoms are present (see section 3).

Can the “diving accident” guideline be used for breath-hold diving?

*If, following a dive, a breath-hold diver develops symptoms of a diving accident consistent with the definition applied herein, this guideline shall be used.*

– Yes: 11/11, no: 0, abstentions: 0

– Strength of consensus: 100% (strong consensus)

There is no clear definition for the term “diving accident,” either nationally or internationally. Both in daily routine and in the literature this term is sometimes used to refer to all medical incidents and events occurring in temporal relation to diving. However, diving incidents need not necessarily be associated with hyperbaric exposure, e.g., myocardial infarction while diving. Likewise in the case of incidents associated with hyperbaric exposure, there is a broad range of relevant disorders, such as barotrauma and submersion pulmonary edema, over and above the diving accidents defined in this guideline.

As a general principle, one should assume that a diving accident has occurred in the event of a medical incident in temporal relation to diving.

A diving accident, according to the definition in this guideline, is characterized by the formation or introduction of gas bubbles in(to) blood and tissues. These processes can lead to decompression sickness. Other terms used in English include “decompression incident” or “decompression injury,” for which the internationally accepted abbreviation is “DCI.” In German, the term “decompression accident” (*Dekompressionsunfall*) is also used. 

Irrespective of the mode in which they develop, diving accidents can be subdivided into:

- Decompression sickness (DCS)

and

**Figure 1:**
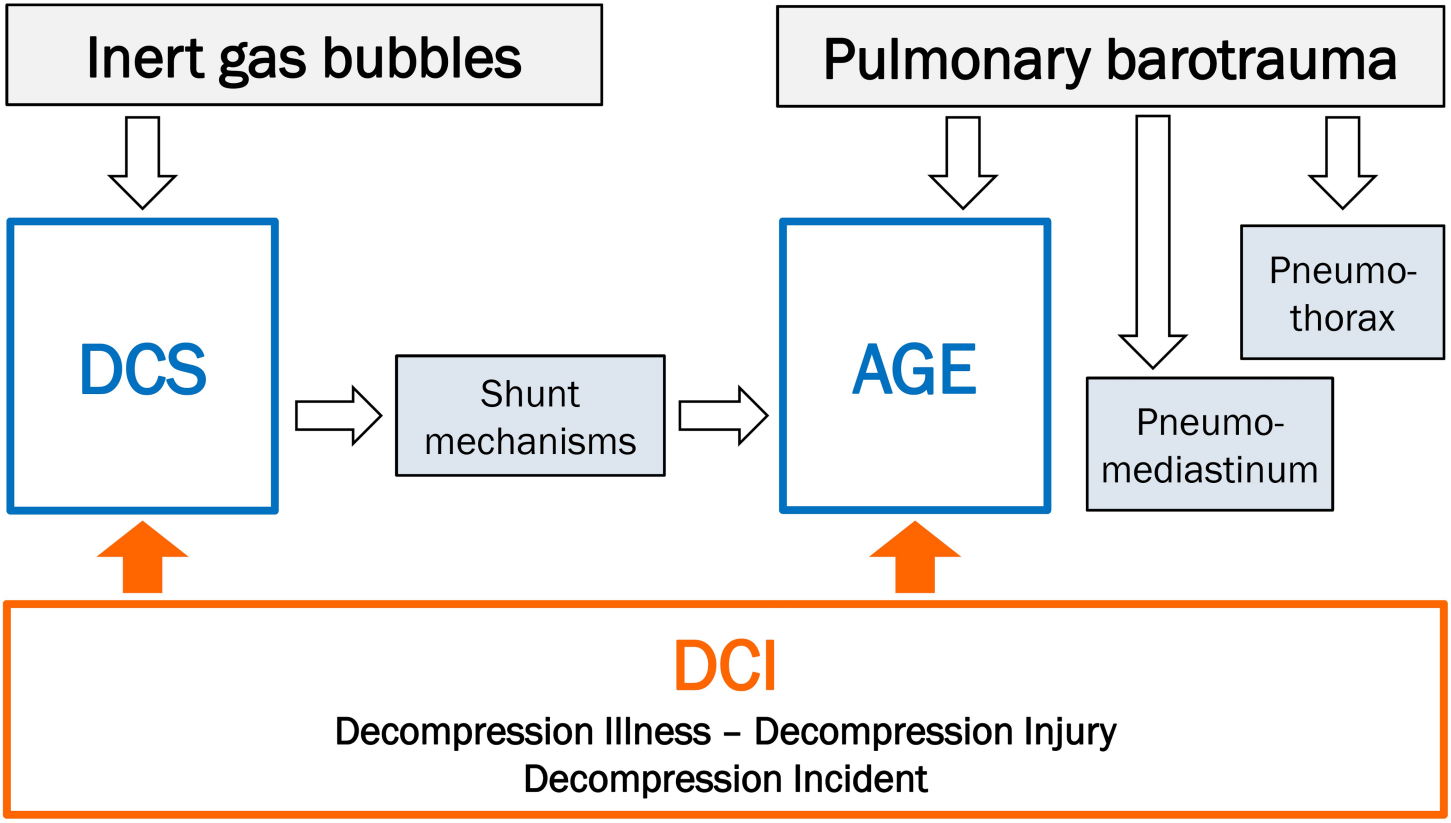
Classification of diving accidents

- Arterial gas embolism (AGE)

(see Figure 1 (Fig. 1): Classification of diving accidents)

### 2.2 Etiology and pathophysiology

#### 2.2.1 Decompression sickness

Bubble formation is assumed to be the primary mechanism of injury in decompression sickness. Divers absorb inert gas (nitrogen when breathing air) into their tissues when they inhale compressed gas during a dive. During ascent the partial pressure of the dissolved gas in tissue can exceed the ambient pressure (oversaturation), resulting in the formation of bubbles in these tissues or in the blood flowing through them.

The resulting venous bubbles, although small (19–700 µm) [4], are very common following dives [5] or rapid exposure to altitude [6]. They are normally filtered through pulmonary capillaries and are asymptomatic. However, venous gas bubbles can reach the arterial circulation by overwhelming the filtering capacity of the pulmonary capillary network or by crossing over through intrapulmonary or intracardiac right-to-left shunts, such as atrial septal defects or patent foramen ovale (PFO).

The presence of a PFO increases the likelihood of decompression sickness in the brain, spinal cord, inner ear, and skin [7], [8] , [9], presumably since tiny arterialized venous gas bubbles that enter the capillaries of oversaturated tissue following a dive grow through inert gas diffusion (nitrogen) [10].

The formation of bubbles in tissue can cause mechanical dysfunction and focal hemorrhage, particularly in the white matter of the spinal cord [11]. Even small intravascular bubbles can have physical sequelae involving inflammatory and thrombogenic responses. Intravascular bubbles can result in impaired regulation of vascular tone, plasma leaks, and hypovolemia [12]. As a result of this mechanism, a large number of venous gas bubbles can injure the pulmonary capillaries and lead to pulmonary edema [13].

#### 2.2.2 Arterial gas embolism

AGE can occur in divers when compressed gas becomes trapped in the lungs and the ambient pressure drops during ascent to the surface. Expansion of the gas results in rupture of the alveolar capillary membrane as well as the entry of gas into the pulmonary vascular system. This can be caused by inadequate expiration of expanding gas from the entire lung or local disease such as bronchial obstruction or bullae.

Even slight differences in pressure on ascent from a depth of only 1 m can be causal here [14].

Large intraarterial bubbles can cause arterial occlusion, ischemia, and infarction. Secondary effects in the brain following bubble-induced ischemia are likely to be similar to processes that occur after a stroke, including the release of excitatory neurotransmitters, oxidative stress, inflammation, and an immune response [15].

### 2.3 Prevention

Despite adhering to all safety standards when diving, it is not possible to completely rule out the occurrence of a diving accident. Prevention involves the diver assuming a high degree of personal responsibility. In order to meet this requirement and to be able to make appropriate decisions, the diver must be aware of the relevant influencing factors and their effects, as well as the options to correct these where necessary.

All dives should be preceded by dive training and dive planning that is appropriate to the dive.

Regular skills training (including self and third-party rescue) and general physical fitness form an important basis for safe diving.

Fundamental to any assessment is a medical history, which depends to a crucial extent on truthful information from the diver, as well as a qualified diving medical examination (“diving fitness”); this consists of a clinical examination and instrument-based examinations (e.g., ECG, stress ECG where appropriate, lung function, otoscopy). In addition to the detection of absolute contraindications (e.g., seizure disorders, impaired cardiovascular performance), a proactive consultation is also a key component of any diving medical examination. This consultation always includes general aspects for all divers as well as individual aspects arising from possible risk factors or examination findings.

The general consultation is complementary to the contents of the diver training and should include, for example, the aspect of dehydration risk (lack of fluid intake, fluid loss through sweating and/or diarrhea, etc.) or information on temperature balance and behavior in the case of transient sicknesses. Depending on the diver being examined, the individual part of the consultation is multi-layered and can include subjects such as behavior in the case of overweight (e.g., ensuring adequate physical fitness and following the “low bubble diving” rule), sea sickness, chronic diseases, and medication, not least depending on the planned dive. In the case of relative contraindications, a discussion should be had with the diver regarding how this increased risk for a diving accident can be reduced by appropriate measures.

The dive itself can be made safer through good and conservative dive planning, e.g., according to the low-bubble diving rule.

Prior to each dive, the diver also needs to assess his or her own health status to determine whether factors that hinder safety are present.

Behavior following a dive can also affect the risk for the occurrence of a diving accident. For example, increased physical exertion (difficult exit from the water or carrying heavy pieces of equipment) or short intervals before subsequent flights increase the risk of bubbles being released and circulated.

## 3 Symptoms and diagnosis

Which examination methods are suitable for the diagnosis, differential diagnosis, and follow-up of a decompression accident?

*All new-onset symptoms after a dive shall be considered as a possible diving accident unless some other mecha* *nism of onset is apparent.*

– Yes: 11/11, no: 0, abstentions: 0

– Strength of consensus: 100% (strong consensus)

*The suspected diagnosis of “diving accident” should be made on the basis of symptoms, taking into consideration the dive and any pre-existing problems or diseases. A physician* *^1^* *trained in diving medicine should be consulted as soon as possible.*

– Yes: 11/11, no: 0, abstentions: 0

– Strength of consensus: 100% (strong consensus)

*Diving accident victims should be closely monitored for the onset of symptoms or worsening of existing symptoms.*

– Yes: 11/11, no: 0, abstentions: 0

– Strength of consensus: 100% (strong consensus)

*Diving accident victims shall undergo in particular a neurological examination as soon as possible. An initial neurological examination shall already be carried out by first aiders, assuming this does not hinder further care.*

– Yes: 10/10, no: 0, abstentions: 1

– Strength of consensus: 100% (strong consensus)

The broad diversity in the clinical picture of DCI hampers diagnosis.

The diagnosis of DCI and any differential diagnoses that may need to be taken into account need to be assessed on the basis of clinical symptoms.

Complementary technical investigations are not required for the diagnosis of DCI. However, these may be needed in order to distinguish between differential diagnoses.

Due to the frequency of neurological symptoms [16], [17], all divers in whom a diving accident is suspected should undergo a neurological examination. A lay examination by first responders according to a predefined examination procedure (see Attachment 1) can enable early recognition of neurological symptoms as well as follow-up documentation of symptom severity.

On completion of a dive, symptoms of a diving accident can rapidly change before and after the initiation of treatment; therefore, follow-up examinations are required.

Which classification is suitable for the assessment of severity of a diving accident?

*The treatment approach differs depending on whether symptoms are mild or severe. Therefore, this guideline classifies diving accident severity according to this classification (see section 3.1 and 3.2).*

– Yes: 10/10, no: 0, abstentions: 0

– Strength of consensus: 100% (strong consensus)

The international literature describes a number of classifications of diving accidents. The best known of these remains the traditional classification that is still used worldwide today, which subdivides decompression accidents into DCS I: bends, pain only, mild, minor symptoms, DCS II: severe, serious, major symptoms, and arterial gas embolism (AGE). Modified classifications that also distinguish between “mild symptoms” and “serious symptoms” have been advocated.

The classification into mild symptoms and severe symptoms used in this guideline differs from the majority of classifications in international use in order to adequately treat patients with apparently “milder” symptoms both consistently and at an early stage, thereby avoiding late sequelae or complications.

This guideline classifies diving accident severity according to the following classification.

### 3.1 Mild symptoms

- Unusual tiredness
- Itching without visible skin changes

### 3.2 Severe symptoms

- Visible spots and changes on the skin
- Tingling (e.g., formication)
- Numbness
- Subcutaneous swelling (lymphatic symptoms)
- Limb pain (bends)
- Pain around the midriff
- Paralysis
- Bladder dysfunction
- Impaired coordination and gait
- Impaired vision, hearing, and speech
- Dizziness
- Nausea
- Impaired consciousness
- Physical weakness
- Difficulty breathing
- Cardiovascular problems (chest tightness, shock)

Which other diving-related health impairments should be taken into consideration in the differential diagnosis of diving accidents?

In addition to decompression sickness and AGE, a number of other diving-related disorders can occur, including:

- Barotrauma to the sinuses, as well as the middle, outer, or inner ear
- Barotrauma to other air-filled cavities in or on the diver’s body (e.g., mask)
- (Tension) pneumothorax
- Pneumomediastinum
- Submersion pulmonary edema
- Alternobaric vertigo
- Drowning accident
- Hypothermia

## 4 Treatment

In the case of diving accidents, diving partners, safety divers, diving group leaders, and diving instructors are usually at the scene to carry out first aid measures.

The success of initial measures as well as the further treatment depends to a crucial extent on first-aid measures being carried out rapidly and correctly.

Requirements [18]:

- Appropriate training completed by all divers
- Availability of emergency equipment tailored to the dive plan
- A diving accident plan (diving emergency plan, telephone numbers)
- Reliable means of communication

### 4.1 First-aid measures

Which measures are first-aiders recommended to take?

**Figure 2:**
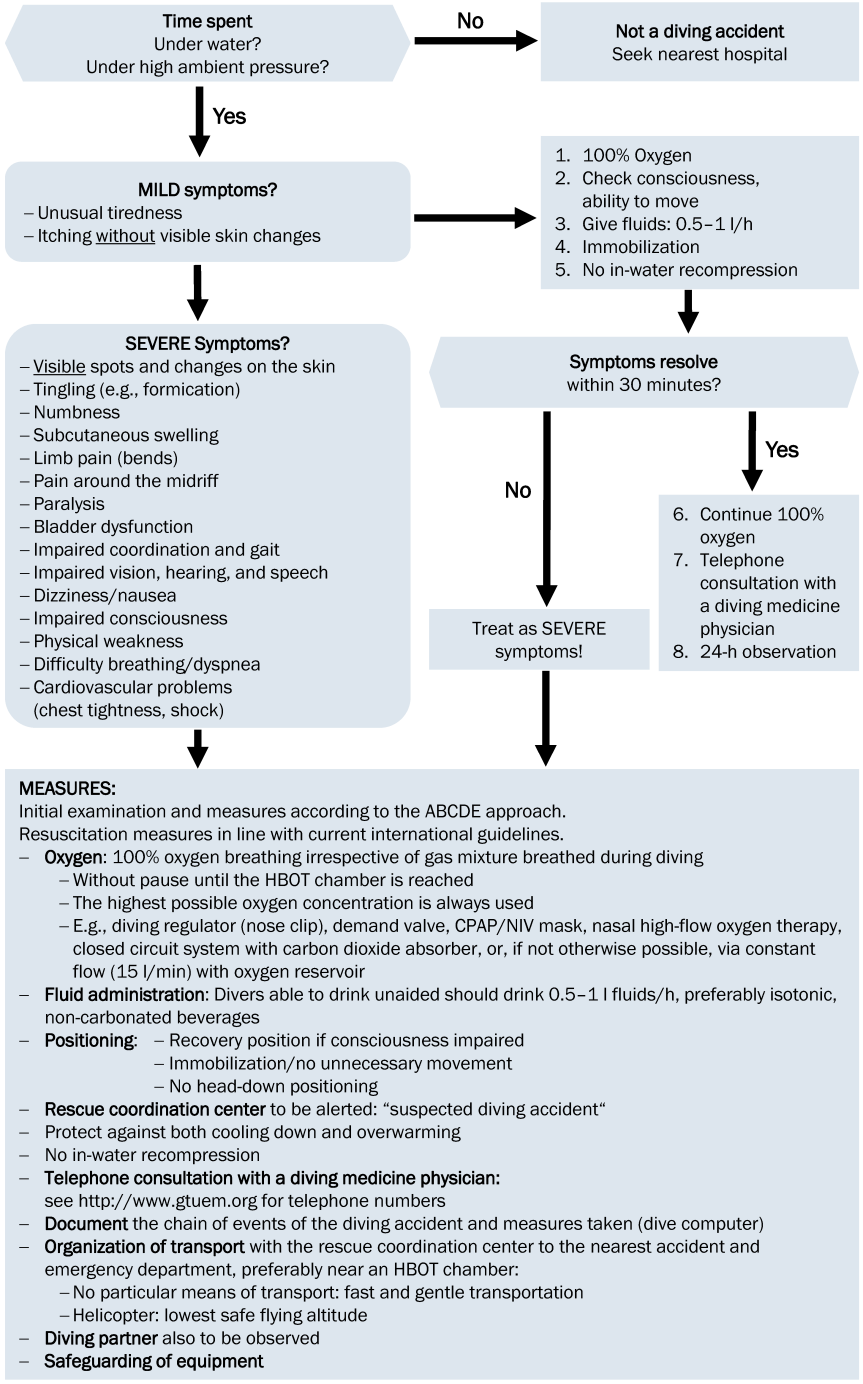
Flow diagram “First aid in diving accidents”

**Measures for mild symptoms** (see Figure 2 (Fig. 2))

- *Immediate breathing of 100% oxygen or breathing gas with the highest available oxygen content irrespective of the gas mix used during diving *[19]*, *[20]* (see section 4.4)*
- *Checking consciousness, ability to move, and perception (e.g., “Basic neurological assessment for divers,” see Attachment 1* *)*
- *Divers that are able to drink unaided should be encouraged to drink 0.5–1 l fluids/h *[18]*, *[21]*, *[22]* (preferably isotonic, non-carbonated beverages/no alcoholic beverages)*
- *Protect against both cooling down and overwarming *[23]*, *[24]
- *No in-water recompression*
- *Continue 100% oxygen breathing until a diving medicine specialist can be consulted, even if the diver is symptom-free within 30 min*
- *Telephone consultation with a diving medicine specialist *[18]* (see section 4.2)*
- *Document the chain of events of the diving accident and measures taken*
- *If symptoms persist unchanged after 30 min or reoccur, treat as severe symptoms*
- *Observe diver for 24 h following resolution of mild symptoms *[18]*, *[25]

*Diving partners may also develop symptoms in the fur* *the* *r c* *ourse. They should be observed for mild or seve* *re sy* *mptoms and, if necessary, included in further di* *a* *g* *n* *o* *s* *tic and therapeutic measures.*

**Measures for severe symptoms** (see Figure 2 (Fig. 2))

*In the case of unconscious divers without identifiable independent breathing, the recommendations on re* *s* *us* *ci* *t* *a* *tion measures according to the current international guidelines apply* *^2^* *.*

- *Cardiopulmonary resuscitation (basic life support)*

*Diving accident-specific first aid*

- *Immediate breathing of 100% oxygen or breathing gas with the highest available oxygen content irrespective of the gas mix used during diving *[26]*, *[27]* (see section 4.4)*
- *Check consciousness, ability to move, and perception (e.g., “Basic neurological assessment for divers,” see Attachment 1* *)*
- *Positioning *[18]*, *[23]*, *[28]*, *[29]*, *[30]*:*
- *Lateral recumbent position if consciousness impaired*
- *Immobilization/no unnecessary movement*
- *No head-down positioning*

– Yes: 10, no: 0, abstentions: 0

– Strength of consensus: 100% (strong consensus)

### 4.2 Telephone consultation with a diving medicine specialist 

A physician^1^ trained in diving medicine should be consulted as to whether hyperbaric oxygen therapy (HBOT) is required and how urgent this is. These decisions generally lie beyond the scope of medical laypersons and physicians without diving medical training.

- National Divers Alert Network (DAN) hotline for Germany and Austria:
  00800 326 668 783 (00800 DAN NOTRUF)
- National DAN hotline for Switzerland (via REGA):
  +41 333 333 333 (or 1414 for calls within Switzerland)
- VDST hotline:
  +49 69 800 88 616
- Naval Medical Institute of the German Navy (SchiffMedInstM):
  +49 431 5409 1441
- aqua med diving hotline:
  +49 421 240 110-10
- International DAN hotline:
  +39 06 4211 8685 or 5685

Please use the code “Diving Accident” for all phone numbers.

An up-to-date list can be found on the GTÜM website (see http://www.gtuem.org).

### 4.3 Measures for medical personnel

Which measures are medical professionals recommended to take?

*Initial examination and measures according to the ABCDE approach.*

*Resuscitation measures shall be performed in line with current international guidelines* *^2^* *:*

- *Advanced life support*
- *Exclusion/treatment of tension pneumothorax*

*Diving accidents can result in drowning accidents, which then require specific treatment.*

*Measures for mild symptoms are the same as those undertaken by first responders.*

**Diving accident-specific measures for severe symptoms** (see Figure 2 (Fig. 2))

- *Immediate breathing of 100% oxygen or breathing gas with the highest available oxygen content ir* *re* *spect* *i* *ve of the gas mix used during diving (see sec* *tio* *n 4* *.4)*
- *Airway management*
  - *In the case of insufficient oxygenation and adequate vigilance, a continuous positive airway pressure noninvasive ventilation (CPAP/NIV) mask or nasal high-flow oxygen therapy should be preferred over intubation for an ongoing neurological assessment*
- *Fluid replacement *
  - *0.5–1 l intravenous fluids/h *[18]*, *[31]* (preferably full electrolyte solution)*
- *Positioning *[18]*, *[23]*, *[28]*, *[29]*, *[30]*:*
  - *Patient positioning according to emergency medical standards*
  - *Patient immobilization/no unnecessary movement*
- *Drugs*
  - *With the exception of oxygen, there are no drugs for which there is clear scientific evidence of effi* *c* *a* *cy in the treatment of diving accidents. All drugs administered as part of advanced life support shall be used in line with the indication.*
- *No in-water recompression*
- *Other measures*
  - *As a basic principle, methods in accordance with emergency medicine standards*
  - *Clinical and neurological examinations to be carried out as soon as possible and during follow-up*
  - *Monitoring*
  - *If necessary, urinary catheter*
  - *Protection against both cooling down and overwarming. In the case of hypothermia, no active rewarming, since this can exacerbate the symptoms of a diving accident*
  - *Telephone consultation with a diving medicine specialist (see section 4.2)*
  - *In the case of severe symptoms, initiate HBOT as rapidly as possible* *^3^*
  - *HBOT is required in the majority of cases, even if treatment initiation is delayed*
  - *Documentation of dive data (dive computer), the course of symptoms, and the treatment measures performed*
  - *Assess whether diving partner also needs to be examined and possibly treated by a physician* *^1^* *trained in diving medicine*

– Yes: 7, no: 0, abstentions: 0

– Strength of consensus: 100% (strong consensus)

– This vote was held with and without members of the guideline group with conflicts of interest regarding the recommendations on HBOT. Strong consensus emerged for the recommendations listed here with and without abstentions (10 of 10).

Are there alternative and/or complementary treatment methods to HBOT (including drugs, statement on in-water recompression [IWR])?

*There are no alternative treatment methods to HBOT* *^3^* *.*

*With the exception of oxygen, there are no drugs for which there is clear scientific evidence of efficacy in the treatment of diving accidents. All drugs administered as part of advanced life support shall be used in line with the indication.*

*IWR should not be performed. This is reserved for professional teams with appropriate training, experience, as well as personnel and equipment if a hyperbaric chamber cannot be reached within a matter of hours in the case of a life-threatening diving accident *[32]*, *[33]*.*

– Yes: 7, no: 0, abstentions: 0

– Strength of consensus: 100% (strong consensus)

– This vote was held with and without members of the guideline group with conflicts of interest regarding the recommendations on HBOT. Strong consensus emerged for the recommendations listed here with and without abstentions (10 of 10).

HBOT has remained unchallenged as a treatment method for diving accidents ever since the first cases were described [34], [35], [36], [37]. With the establishment of oxygen therapy during this treatment, HBOT represents the worldwide treatment standard today [38], [39], [40], [41]. Delayed initiation of recompression therapy, especially if longer than 6 h, increases the risk of irreversible damage [25], [42], [43], [44], [45].

### 4.4 Oxygen therapy/oxygen administration (normobaric oxygenation)

Which method of oxygen administration should be preferred?

*For the administration of oxygen, the method that delivers the highest proportion of oxygen available for breathing or ventilation of the victim should be selected. The conservation of resources plays a secondary role here.*

– Yes: 10, no: 0, abstentions: 0

– Strength of consensus: 100% (strong consensus)

How should oxygen be administered?

**Oxygen administration (normobaric oxygenation)**

*The causal treatment of diving accidents consists of breathing pure oxygen *[46]*, *[47]*, *[48]*, *[49]*, *[50]*, *[51]*, *[52]* (FiO_2_ 1.0, “100%”).*

*Even in the event that the O* *_2_* *supply is limited, O* *_2_* *in the highest available concentration shall always be administered, accepting that transport may need to be completed with air breathing.*

*Time delays need to be avoided. Immediate 100% oxygen breathing is irrespective of the gas mix used during diving.*

- *If the victim’s* *independent breathing is sufficient* *, respiration of 100% oxygen (verify addition of oxygen) with:*
  - *Diving regulator (nose clip) *[53]
  - *Demand valve *[54]
  - *CPAP/NIV mask (consider risk in suspected pneumothorax)*
  - *Nasal high-flow (NHF)/high-flow (HFOT)/high-flow nasal cannula (HFNC) oxygen therapy *[53]
  - *Closed circuit system with carbon dioxide absorber*
  - *If no better systems are available, via constant flow (15–25 l/min, non-rebreathing mask with oxygen reservoir)*
- *If the victim’s* *independent breathing is not sufficient* *, airway management in accordance with emergency medicine standards and* *artificial respiration* *(assisted or controlled) with 100% oxygen via:*
  - *Exclusion/treatment of tension pneumothorax*
  - *CPAP/bi-level PAP (BiPAP) (consider risk in sus* *pe* *c* *ted pneumothorax)*
  - *Closed circuit system with carbon dioxide absorber*
  - *If no better systems are available, bag valve mask with demand valve or oxygen reservoir bag and constant flow (at least 15 l/min)*

*The administration of 100% oxygen shall be continued without pause until the HBOT chamber is reached.*

– Yes: 10, no: 0, abstentions: 0

– Strength of consensus: 100% (strong consensus)

### 4.5 Transport

Which means of transport are suitable for diving accident victims (vehicle, helicopter, aircraft, boat)?

*There is no general preference for a particular means of transport. Bearing in mind the total time required for transport, the fastest and most gentle means of transport shall be used.*

- *Helicopter (lowest safe flying altitude)*
- *Ground-based rescue vehicles (risk posed by a further drop in pressure when driving over mountain passes)*
- *Boat*

– Yes: 10, no: 0, abstentions: 0

– Strength of consensus: 100% (strong consensus)

*All available information, such as documentation of dive data (diving computer), course of symptoms, and previous treatment measures shall remain with the diving accident victim.*

– Yes: 10, no: 0, abstentions: 0

– Strength of consensus: 100% (strong consensus)

- Organization of means of transport via the rescue coordination center
- Transport destination: nearest suitable and accessible accident and emergency department, preferably near an HBOT chamber that meets the standards set out by the GTÜM.

#### 4.5.1 Treatment during transportation

Clinical and orienting neurological examination to be regularly repeated.

### 4.6 Hyperbaric oxygen therapy

When is HBOT indicated following a diving accident?

*The initial HBOT treatment shall take place as soon as possible. Even delayed treatment initiation (even after days) can achieve an improvement in symptoms *[45]*, *[55]*, *[56]*, *[57]*, *[58]*.*

*The indication for HBOT is met in the case of:*

- *Mild symptoms that do not resolve even after 30 min breathing 100% pure oxygen*
- *Severe symptoms (HBOT always indicated)*

– Yes: 7, no: 0, abstentions: 0

– Strength of consensus: 100% (strong consensus)

– This vote was held with and without members of the guideline group with conflicts of interest regarding the recommendations on HBOT. Strong consensus emerged for the recommendations listed here with and without abstentions (10 of 10).

#### 4.6.1 Measures prior to initial HBOT treatment

Imaging is not routinely required. If pneumothorax is suspected, imaging shall be performed.

- Chest X-ray
- Ultrasound or
- Computed tomography

If a further diagnostic work-up according to emergency medicine standards is urgently indicated to rule out other causes of the victim’s condition, the delay to HBOT should be as short as possible.

The following measures may be required:

- Pleural drainage
- Paracentesis in unconscious patients if this can be performed by an expert without a time delay
- Urinary catheter

#### 4.6.2 Treatment tables

Which treatment tables should be used?

**Figure 3:**
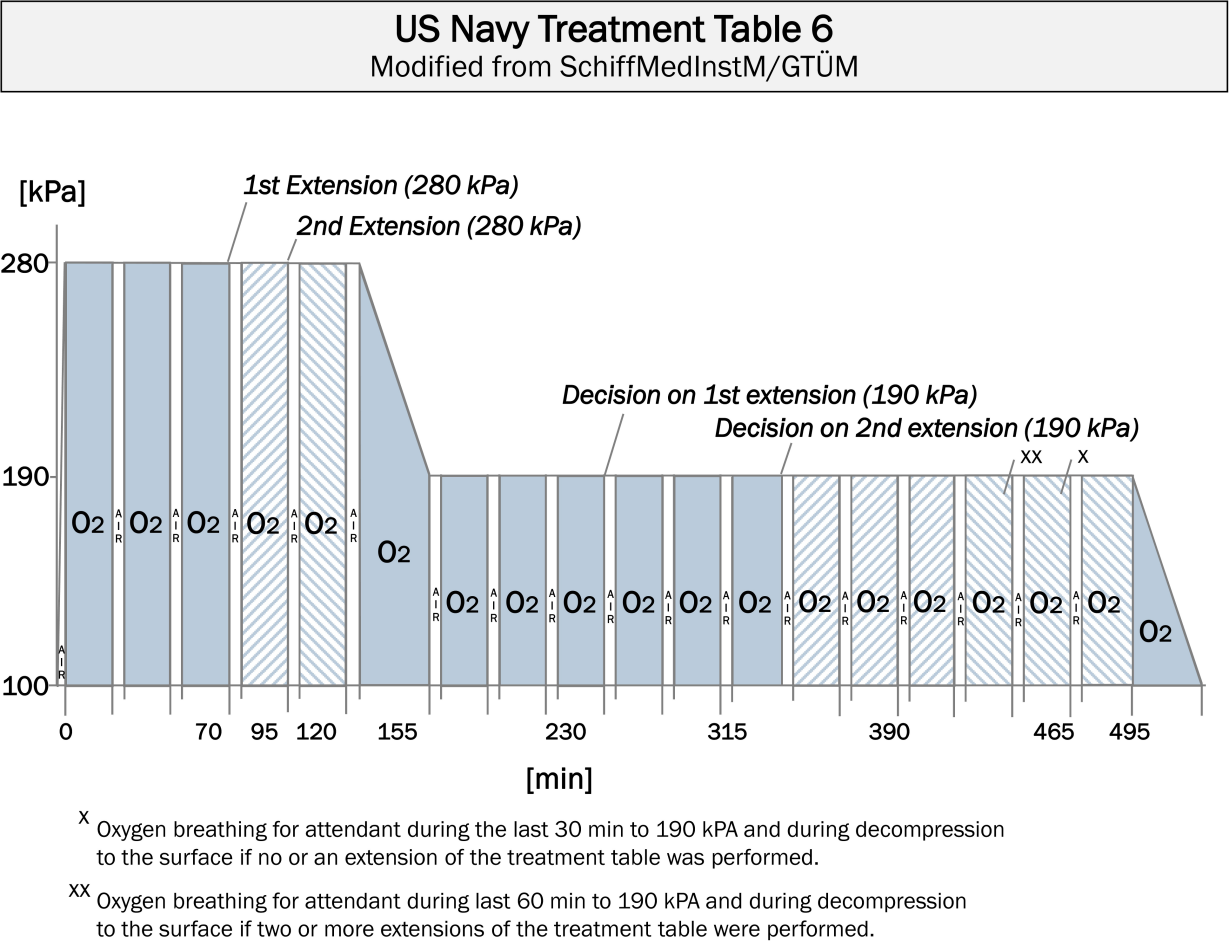
Modified “US Navy Treatment Table 6”

*The standard treatment table is the ‘US Navy Treatment Table 6’ *[41]*, *[45]*, *[59]*, *[60]*, *[61]*, *[62]* or modifications thereof with an initial treatment pressure of 280 kPa (see Figure 3 *(Fig. 3)*).*

– Yes: 7, no: 0, abstentions: 0

– Strength of consensus: 100% (strong consensus)

– This vote was held with and without members of the guideline group with conflicts of interest regarding the recommendations on HBOT. Strong consensus emerged for the recommendations listed here with and without abstentions (10 of 10).

Does the treatment method depend on the breathing gas used?

*The standard ‘US Navy Treatment Table 6’ shall be used for all diving accidents, irrespective of the breathing gas used by the diving accident victim.*

– Yes: 10, no: 0, abstentions: 0

– Strength of consensus: 100% (strong consensus)

HBOT can be shortened in the case of complete resolution of the symptoms listed below within the first 10 min of hyperbaric oxygenation at 280 kPa.

- Constitutional or nonspecific symptoms: marked tiredness
- Cutaneous symptoms: skin changes, skin bends
- Lymphatic symptoms: local swelling
- Musculoskeletal symptoms: joint and limb pain
- Mild subjective peripheral neurological sensory disturbances without identifiable pathological findings

In such cases, treatment can be shortened in line with ‘US Navy Treatment Table 5’ or similar tables. However, it is essential that no additional symptoms are (or have been) present.

If complaints or symptoms fail to (completely) resolve under hyperbaric oxygenation, the initial HBOT treatment is prolonged. At a treatment pressure of 280 kPa, a maximum of two 25-min extensions (20 min oxygen breathing and 5 min air breathing) are performed; at a treatment pressure of 190 kPa, a maximum of two 75-min extensions (3x 20 min oxygen breathing and 3x 5 min air breathing) are also performed.

- If the treated diver is not almost symptom-free after 60 min (3x 20 min) of oxygen breathing at the initial treatment pressure of 280 kPa, an initial extension of 20 min oxygen breathing and 5 min air breathing is performed at this treatment pressure.
- If the treated diver is not almost symptom-free after 80 min (4x 20 min) oxygen breathing at 280 kPa, a second extension of 20 min oxygen breathing and 5 min air breathing is performed. Decompression is then performed to 190 kPa according to ‘US Navy Treatment Table 6‘.
- If the treated diver is not almost symptom-free after 60 min (3x 20 min) oxygen breathing at a treatment pressure of 190 kPa, a third extension of a further 60 min (3x 20 min) oxygen breathing and 15 min (3x 5 min) air breathing is then performed after a total of 120 min (6x 20 min) oxygen breathing at this pressure.
- If the treated diver is not almost symptom-free after 60 min (3x 20 min) oxygen breathing at a treatment pressure of 190 kPa, a third extension of a further 60 min (3x 20 min) oxygen breathing and 15 min (3x 5 min) air breathing is then performed after a total of 120 min (6x 20 min) oxygen breathing at this pressure. After a total of 240 min oxygen breathing at 190 kPa, decompression to ambient pressure is then performed according to ‘US Navy Treatment Table 6‘.

Other treatment tables, in particular tables with longer treatment times and higher treatment pressures, as well as mixed gas and saturation treatment tables, should be reserved for centers and personnel with special experience, knowledge, and suitable equipment that allow them to deal with adverse events and outcomes. Oxygen-enriched breathing gas mixtures are to be used for all treatment tables. 

**Figure 4:**
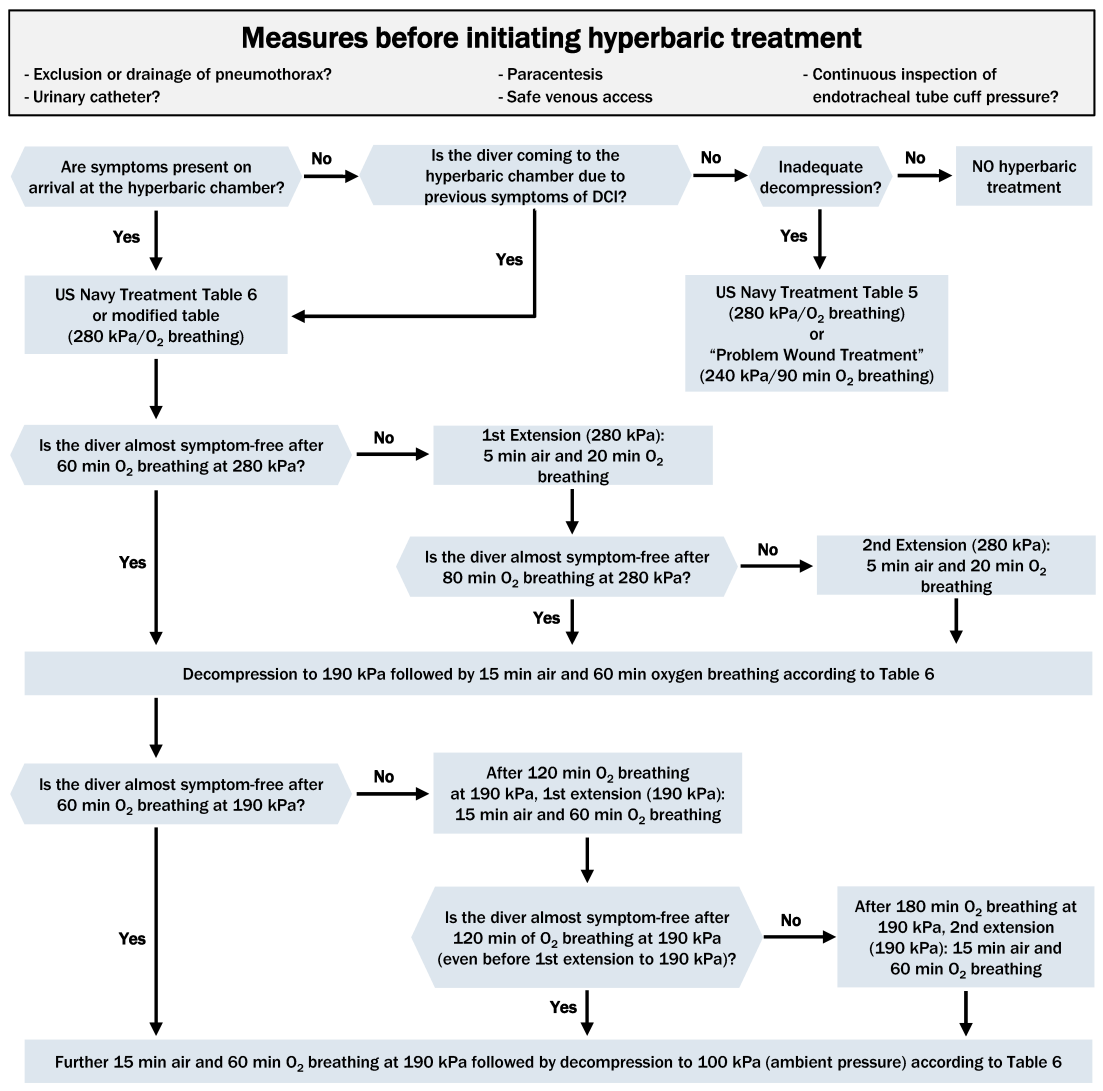
Flow diagram “Initial hyperbaric oxygen treatment in diving accidents”

If HBOT is indicated in the case of inadequate decompression without symptoms, shorter treatment tables are possible, for example, ‘US Navy Treatment Table 5’ or the ‘Problem Wound Treatment Protocol’ (see Figure 4 (Fig. 4)).

If initial HBOT fails to achieve an improvement, the differential diagnosis needs to be reviewed.

#### 4.6.3 Measures during initial HBOT

- Neurological check-ups, e.g., during air breathing phases, should always be repeated before deciding whether extensions of the treatment table may be necessary (documentation!).
- Repeated clinical examination and lung auscultation (pneumothorax? bilaterally equal ventilation?), particularly following pressure drops in the treatment table
- Regular inspection of all sealed air-filled cavities in medical devices (e.g., endotracheal tube cuff, infusion, drip chamber, blood pressure cuff), always before and during pressure reductions in the treatment table
- As a basic principle, methods in accordance with emergency medicine standards
- Fluid balancing
- With the exception of oxygen, there are no drugs for which there is clear scientific evidence of efficacy in the treatment of diving accidents.
- Document all measures performed for transfer to continuing-care providers/physicians.

#### 4.6.4 Measures following initial HBOT

What treatment do patients receive between HBOT treatments?

*All patients should remain under observation for at least 24 h following the initial HBOT treatment.*

*If the patient is in a critical condition, intensive care may be necessary.*

*Between HBOT treatments, supplemental oxygen is administered only if blood oxygen is low (hypoxemia). Ele* *v* *a* *ted oxygen levels are not targeted.*

*Further treatment is carried out according to the clinical picture and in accordance with the specialties involved.*

– Yes: 10, no: 0, abstentions: 0

– Strength of consensus: 100% (strong consensus)

- Earliest possible start of intensive specific treatment and rehabilitation measures if feasible to accompany HBOT.
- There is no evidence for a benefit from physiotherapy during HBOT versus physiotherapy alone between HBOT treatments.
- Pharmacological and further treatment is carried out according to the clinical picture and in accordance with the specialties involved.

#### 4.6.5 Further HBOT treatments

Are follow-up HBOT treatments recommended?

*If symptoms are still present following the initial HBOT treatment, a follow-up session should take place within 24 h.*

– Yes: 7, no: 0, abstentions: 0

– Strength of consensus: 100% (strong consensus)

– This vote was held with and without members of the guideline group with conflicts of interest regarding the recommendations on HBOT. Strong consensus emerged for the recommendations listed here, with and without abstentions (10 of 10).

- At least 1x daily HBOT, e.g., according to the Problem Wound Treatment Protocol [33].
- If severe neurological symptoms persist, a second HBOT treatment can also be considered according to the standard ‘US Navy Treatment Table 6’.
- Other treatment tables should be reserved for centers and personnel with special experience, knowledge, and suitable equipment that allow them to deal with adverse events and outcomes.

#### 4.6.6 Intervals between HBOT treatments

- No more than 24 h, but no more than two sessions within 24 h

#### 4.6.7 Further diagnostic work-up/follow-up examinations according to clinical symptoms

- Magnetic resonance imaging (MRI)
- Computed tomography (CT)
- Specialist neurological consultations (regularly)
- Further specialist medical consultations according to symptoms and organ systems affected

#### 4.6.8 Decision-making on discontinuation of HBOT

- HBOT can be discontinued following complete and lasting freedom from symptoms.
- If, after several treatments, there is no further improvement in symptoms over 3–5 days after an initial improvement under continued treatment, HBOT should be discontinued.

### 4.7 Treatment of children and adolescents

What is the treatment for children and adolescents?

*Diving accidents according to the definition in this guideline are rarer in children and adolescents than in adults. Their treatment does not differ significantly from that of adults.*

*Treatment primarily consists of high-dose oxygen administration, and if necessary, timely HBOT. Fluid and drug dosage shall be age- and weight-adjusted.*

*Suitable and tailored equipment shall be available to perform treatment.*

*The treatment of children and adolescents should be carried out in an age-dependent manner in collaboration between a physician experienced in pediatric (intensive) care and the HBOT center *[63]*.*

– Yes: 10, no: 0, abstentions: 0

– Strength of consensus: 100% (strong consensus)

### 4.8 Transfer (secondary transport)

If symptoms persist following initial HBOT, further treatments may need to be carried out within 24 h if the diagnosis is confirmed. If on-site inpatient medical care is not available between HBOT treatments, the patient must be transported to an appropriately equipped treatment center^3^. The means of transport is chosen taking into account the patient’s status, the distance and time to the center, and the possible “means of transport.”

- Helicopter
- Air ambulance
- Passenger aircraft
- Boat
- Land-based rescue vehicles

There is no reliable data to support a blanket requirement for transport under 1-bar conditions for secondary transportation. Aircraft with normal cabin pressure (e.g., 0.8 bar absolute) are much faster and easier to organize.

There is evidence that DCI recurrences following HBOT are more common during or after a flight than in patients that do not fly. There is also evidence that the onset of symptoms of higher severity is not expected during a flight and that treatment prospects are not worsened.

Transport by air at normal cabin pressure (e.g., 0.8 bar absolute) does not represent a fundamental obstacle to the transportation of patients following HBOT.

The decision to use this means of transport should be made based on: a) the previous course of decompression sickness and b) the severity of ongoing symptoms. There are no uniform international recommendations specifying the time interval after which, and after how many HBOT treatments, DCI patients should be transported by air and at what cabin pressure. These decisions should be made on a case-by-case basis in consultation with experienced diving physicians.

#### 4.8.1 Medical care during secondary transport

The need for and extent of medical care during transportation depends on the severity of the clinical picture.

- Procedures according to emergency medicine/intensive care standards
- Oxygen breathing must be possible
- Fluid balancing
- Clinical and neurological monitoring
- Documentation, e.g., emergency physician/intensive care transport protocol
- Patients with no or minimal residual symptoms following primary treatment can be transported on a normal scheduled flight.

## 5 Rehabilitation

Which rehabilitation measures are recommended following a decompression incident?

*Following a diving accident, the specialty and form (outpatient, inpatient) of a rehabilitation measure should be determined on the basis of the specific functional impairment and its extent.*

– Yes: 10, no: 0, abstentions: 0

– Strength of consensus: 100% (strong consensus)

- Diving accidents can lead to neurological, psychological, cardio-circulatory, pulmonary, constitutional, and orthopedic impairments [64], [17]. Neurological symptoms are often the cause of lasting physical impairments.
- Extent and type, or extent of functional impairment, are central to the choice of rehabilitation measure.
- There are neither specific rehabilitation programs for diving accident patients nor studies on rehabilitation programs for diving accident victims.
- Type, duration, and intensity of rehabilitation measures following a diving accident are based on comparable disorders of other etiology.

## 6 Fitness to dive following a diving accident

How should fitness to dive be assessed following a diving accident?

*The assessment of fitness to dive following a diving ac* *cide* *nt shall be made in accordance with the recommen* *da* *tions of the national and international specialist socie* *t* *i* *es for diving medicine or, where applicable, the rele* *v* *a* *nt national legislation.*

– Yes: 10, no: 0, abstentions: 0

– Strength of consensus: 100% (strong consensus)

The precondition for a re-assessment of fitness to dive is the definitive completion of diving accident therapy and the stability of the treatment outcome, even in the case of residual effects.

Any re-assessment of fitness to dive shall be carried out by an experienced physician^1^ with advanced training in diving medicine. They are additionally required to have practical experience in the treatment of diving accidents.

For commercial divers, special national legal provisions apply, including the associated occupational medical screening and fitness-to-dive tests.

## 7 Quality management

Guidelines are intended to form a good information basis, provide orientation and, as decision-making aids, promote the transfer of the best available evidence from clinical studies and the professional expert consensus into everyday care [65].

Guidelines can also support concrete decision-making and action processes, particularly in rare emergencies.

Metrics will be developed and recorded in order to evaluate the application and verify the implementation of this guideline. Taking into consideration the treatment workflow, parameters are to be defined that evaluate process, structure, and, if necessary, outcome quality.

In the following, the guideline group has drawn up proposals for indicators and parameters that will be further developed and whose application will become established following the publication of this guideline.

To this end, it would be possible in principle to use routine administrative data, e.g., from the data sets of the DIVI emergency physician protocol and emergency admission register [66], as well as, if necessary, data from a national HBOT registry in Germany that is to be developed.

### 7.1 Pre-hospital performance indicators

Taking into consideration the treatment workflow, parameters have been described and performance indicators formulated (see Table 1 (Tab. 1)).

1. 100% oxygen breathing in the case of a suspected diving accident
  → “start oxygen”
  [time interval: diagnosis to initiation of oxygen therapy]
2. Fluid replacement 0.5–1 l fluids/h intravenously
  → “start fluid”
  [time interval: diagnosis to initiation of fluid replacement]

### 7.2 In-hospital performance indicators

Treatment in the emergency department begins with the initial medical assessment and ends with the transfer or discharge of a patient from the emergency department.

If a diving accident is diagnosed in a patient,

1. symptoms shall be documented at the time of admission, progress documented during emergency room treatment, and symptoms documented at the time of discharge/transfer.
  → “documentation”
  [documentation of symptoms]
2. the highest possible oxygen concentration shall be initiated or continued without delay.
  → “start oxygen”
  [time interval: diagnosis to initiation of oxygen therapy]
3. HBOT shall be performed if there are signs of a severe diving incident.
  → “field to hbot time”
  → “hospital to hbot time”
  [time intervals to initiation of HBOT]

### 7.3 Post-inpatient performance indicators

If a patient is transferred with residual effects following a diving accident, the transfer report should indicate the need for rehabilitation measures and a further, post-inpatient follow-up examination.

1. Patients with residual effects following a diving accident shall be examined for sequelae for 4–6 weeks.
  → “outcome”

### 7.4 Update planning

The application and implementation of the guideline shall be evaluated prior to its update.

## Notes

^1^ Qualifications should at least correspond to the continuing medical education content of the “Diving Medicine Physician”, see http://www.gtuem.org, http://www.suhms.org, or http://www.edtc.org.

^2^ European Resuscitation Council (ERC) guidelines on advanced life support, see https://www.erc.edu.

^3^ Directory of hyperbaric treatment chambers in Germany, Austria and Switzerland, see https://www.gtuem.org.

## Abbreviations

- ABCDE: Airway, breathing, circulation, disability, environment/exposure
- AGE: Arterial gas embolism
- AWMF: Association of the Scientific Medical Societies in Germany (*Arbeitsgemeinschaft der Wissenschaftlichen Medizinischen Fachgesellschaften*)
- BDA: Professional Association of German Anaesthesiologists (*Berufsverband Deutscher Anästhesisten*)
- CPAP: Continuous positive airway pressure
- DAN: Divers Alert Network
- DCI: Decompression illness, decompression incident, decompression injury
- DCS: Decompression sickness
- DGAI: German Society of Anaesthesiology and Intensive Care Medicine (*Deutsche Gesellschaft für Anästhesiologie und Intensivmedizin*)
- DGAUM: German Society for Occupational and Environmental Medicine (*Deutsche Gesellschaft für Arbeitsmedizin und Umweltmedizin*)
- DIVI: German Interdisciplinary Association for Intensive Care and Emergency Medicine (*Deutsche Interdisziplinäre Vereinigung für Intensiv- und Notfallmedizin*)
- DLRG: German Life-Saving Society (*Deutsche Lebens-Rettungs-Gesellschaft*)
- DRK: German Red Cross (*Deutsches Rotes Kreuz*)
- GTÜM: German Diving and Hyperbaric Medical Society (*Gesellschaft für Tauch- und Überdruckmedizin*)
- HBOT: Hyperbaric oxygen therapy
- HFNC: High-flow nasal cannula
- HFOT: High-flow oxygen therapy
- IWR: In-water recompression
- NHFT: Nasal high-flow therapy
- NIV: Non-invasive ventilation
- PFO: Patent foramen ovale
- SchiffMedInstMNaval: Medical Institute of the German Navy (*Schifffahrtmedizinisches Institut der Marine*)
- SUHMSSwiss: Underwater and Hyperbaric Medical Society (*Schweizerische Gesellschaft für Unterwasser- und Hyperbarmedizin*)
- VDD: Association of German Hyperbaric Treatment Centers (*Verband Deutscher Druckkammerzentren*)
- VDST: German Recreational Divers Association (*Verband Deutscher Sporttaucher*)

## Guideline report

The methodological approach to the development of the guideline and, in particular, the management of potential conflicts of interest is presented in the guideline report.

This is freely available online, e.g., on the website of the Association of the Scientific Medical Societies in Germany (AWMF) [67].

## Competing interests

See Attachment 2

## Supplementary Material

Neurological assessment for divers

Conflicts of interest

## Figures and Tables

**Table 1:**
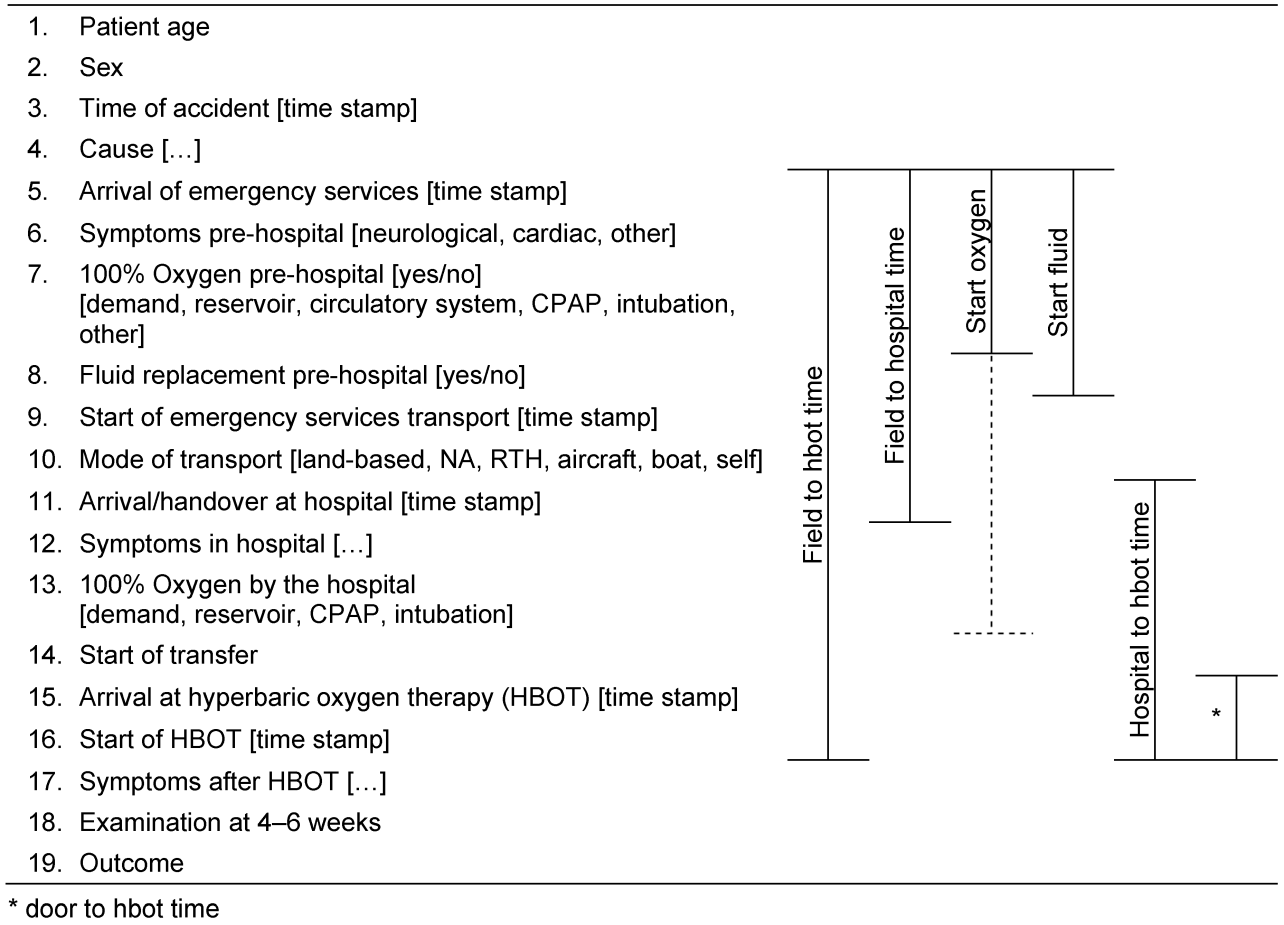
Parameters of the care workflow with performance indicators for process quality
