# Supplementary material for: S2k guideline for diving accidents
Source: Ger Med Sci. 2023 Mar 3;21:Doc01. doi: 10.3205/000315 (PMC10073983; doi:10.3205/000315)
Supplement: Neurological assessment for divers [file GMS-21-01-s-001.pdf]

Neurological Assessment for Divers

Name of the patient: ____

Date: ______________________ **Name of the examiner:** ________________________________

**Examination / Item**	check 1<br>time:<br>_______:_______	check 2<br>time:<br>_______:_______
**1. Level of consciousness**		
Approach the patient verbally, try to shake up an unresponsive patient.<br>→ Is the Patient alert and responsive?	☐ yes ☐ no	☐ yes ☐ no

If not alert and responsive, illustrate further:

☐ The patient is somnolent.

☐ Patient is unresponsive. Check breathing and **prepare for CPR**. Postpone neurological assessment until patient gains consciousness.

**2. Mental Orientation**<br>→ Is the patient able to state the current month and current location correctly?	☐ yes ☐ no	☐ yes ☐ no
**3. Cooperation**<br>→ Is the patient able to follow two given tasks correctly?<br>*(Request: Close your eyes, clench your fist))*	☐ yes ☐ no	☐ yes ☐ no
**4. Speech**<br>→ Is the patient able to identify two simple objects (e.g., pencil, watch, glasses, …) correctly?	☐ yes ☐ no	☐ yes ☐ no
Does the patient speak clearly and with good enunciation/articulation?<br>*(Ask the patient to repeat a given sentence. Can you initiate a clear and structured conversation?)*	☐ yes ☐ no	☐ yes ☐ no
**5. Vision**<br>→ Is the patients vision inhibited?	☐ yes ☐ no	☐ yes ☐ no

If no; if applicable, illustrate the sight disorder (e.g., diplopic images, blurred vision, …):

____

____

**6. Facial Expression**<br>*Ask the patient to bare their teeth.*<br>→ Is the motor response of the face bilaterally equal?<br>*(No: Face and/or mouth react asymmetric.)*	☐ yes ☐ no	☐ yes ☐ no
**7. Motor Activity**<br>*Perform a pronator drift test. Ask the patient to stretch out their arms horizontally, palms up.*<br>→ Is the patient able to hold both arms steady for at least ten seconds?	☐ yes ☐ no	☐ yes ☐ no

If no: Which arm cannot be lifted or hold steadily?

☐ right arm pronates or drops ☐ left arm pronates or drops

Please, turn the page!

Ask the patient in supine position to individually lift each extended leg (approx. 45°).

→ Is the patient able to hold each leg steady? ☐ yes ☐ no ☐ yes ☐ no

If no: Which leg cannot be lifted or hold steadily?

☐ right leg cannot be lifted or is dropped ☐ left leg cannot be lifted or is dropped

8. Sensitivity

Asses the patient for pain, numbness, or paraesthesia.

→ Is the patient without symptoms? ☐ yes ☐ no ☐ yes ☐ no

If no: please illustrate each complaint.

____

____

9. Coordination

Ask the patient to fully extend their right arm, close their eyes and then touch their nose with the tip of their index finger. Repeat for left arm.

→ Is the movement smooth and precise? Does the patient tap the nose? ☐ yes ☐ no ☐ yes ☐ no

Asses for **vertigo**. → Is the patient without symptoms? ☐ yes ☐ no ☐ yes ☐ no

10. Gait and Balance

Avoid any risk of falling! Execute only if patients general condition allows for standing up!

→ Is the patient able to stand still with open eyes/closed eyes? Is the patient standing without sway and tumbling motion? ☐ yes ☐ no ☐ yes ☐ no

→ When walking, does the patient present a normal gait pattern? ☐ yes ☐ no ☐ yes ☐ no

Notes:

____

____

____

____

____

____

VDST
www.vdst.de
Medizinische
NOTFALL-HOTLINE
für VDST-Mitglieder
+49 69 800 88 616

Execution of the 'Neurological Assessment for Divers'

- The patient passes this assessment when all items are answered with '*yes*'.
- A passed assessment does not rule out a diving accident. When the slightest chance of a diving accident is present, react accordingly and follow local procedures.
- The '*Neurological Assessment for Divers*' must not delay the initiation of proper treatment and alerting emergency medical services. (emergency call, CPR and providing normobaric oxygen therapy are paramount!). Proceed with neurological assessment while oxygen is provided.
- This assessments aim is to identify and document neurological symptoms as well as reporting on its development over time. Repeat assessment at short notice (e.g., after 30, 60, 90 minutes) and report on any changes (e.g., subsiding symptoms after normobaric oxygen therapy).
- This document remains with the patient and medical professionals (e.g., hand-over to paramedics, clinicians).
- Item one through nine are assessed in a supine position or with elevated upper body (Fowler's Position). Item 10: Perform only if the patient feels comfortable and safe standing and walking and has mild or minor symptoms.
- Further information on scuba diving accidents and first aid: VDST emergency hotline, 'Leitlinie Tauchunfall'.
