# Supplementary material for: S2k guideline for diving accidents
Source: Ger Med Sci. 2023 Mar 3;21:Doc01. doi: 10.3205/000315 (PMC10073983; doi:10.3205/000315)
Supplement: Conflicts of interest [file GMS-21-01-s-002.pdf]

Declaration of interests and management of conflicts of interest
S2k Guideline Guideline for Diving Accidents 2022-2027

The following is a tabular summary of the declarations of interest, as well as the results of the conflict of interest assessment and actions that were decided upon by the guideline group after discussion of the issues and implemented at the consensus conference.

	Consultant or expert activity	Cooperation on a scientific advisory board	Paid lecturing or training activities	Paid authorship or coauthorship	Research projects/conducting clinical studies	Ownership interests (patent, copyright, share ownership)	Indirect interests	Guideline topics affected by COI<br>Classification in terms of relevance<br>Impact
Björn Jüttner	No	No	Instructor for the training of diving and hyperbaric chamber physicians	No	No	No	Mandate holder of the GTÜM, guideline coordinator<br>Employer: Hannover Medical School<br>Member: DGAI, GRC, EUBS, DIVI	Diving and hyperbaric medicine<br>No relevance<br>No restriction on function in the guideline group
Christian Wölfel	No	No	Instructor for the training of diving and hyperbaric chamber physicians	author of professional journals	No	No	Mandate holder of the SUHMS<br>Employer: Spital Region Oberaargau AG, Switzerland	Diving and hyperbaric medicine<br>No relevance<br>No restriction on function in the guideline group
Claudio Camponovo	No	No	Instructor for the training of diving and hyperbaric chamber physicians	No	No	No	Acting Mandate holder of the SUHMS<br>Employer: Hospita Suisse Anesthesia Care, Switzerland<br>Member: SSAPM, SUHMS (President)	Diving and hyperbaric medicine<br>No relevance<br>No restriction on function in the guideline group
Holger Schöppenthau	No	No	Instructor for the training of diving and hyperbaric chamber physicians	book contribution: diving and hyperbaric medicine	No	No	Mandate holder of the DIVI<br>Employer: BGU Murnau, Medical Director, Hyperbaric Oxygen Therapy<br>Member: GTÜM	Diving and hyperbaric medicine<br>Moderate restriction on in level 2 guideline group (abstention on HBOT recommendations)
Johannes Meyne	No	No	Instructor for the training of diving and hyperbaric chamber physicians	No	No	No	Mandate holder of the VDST<br>self-employed neurologist	Diving medicine<br>No relevance<br>No restriction on function in the guideline group

	Consultant or expert activity	Cooperation on a scientific advisory board	Paid lecturing or training activities	Paid authorship or coauthorship	Research projects/conducting clinical studies	Ownership interests (patent, copyright, share ownership)	Indirect interests	Guideline topics affected by COI<br>Classification in terms of relevance<br>Impact
Konrad Meyne	No	No	No	No	No	No	Acting Mandate holder of the VDST	No<br>No relevance<br>No restriction on function in the guideline group
Carmen Wohlrab	No	No	No	No	No	No	Mandate holder of the Federal Armed Forces<br>Employer: Federal Armed Forces, diving and hyperbaric physician	No<br>No relevance<br>No restriction on function in the guideline group
Henning Werr	No	No	No	No	No	No	Acting Mandate holder of the Federal Armed Forces<br>Employer: Federal Armed Forces, diving and hyperbaric physician<br>Member: GTÜM	No<br>No relevance<br>No restriction on function in the guideline group
Till Klein	No	No	Instructor for the training of diving and hyperbaric chamber physicians	No	No	No	Mandate holder of the VDD<br>Employer: St.-Antonius-Hospital, Eschweiler<br>Member: GTÜM<br>Freelance physician, HBO-Zentrum Euregio Aachen	Diving and hyperbaric medicine<br>Moderate restriction on in level 2 guideline group (abstention on HBOT recommendations)
Giso Schmeißer	No	No	No	No	No	No	Mandate holder of the DGAUM<br>Employer: Deutsche Gesetzliche Unfallversicherung<br>Member: VdBW	No restriction on function in the guideline group
Karsten Theiß	No	No	Instructor for the training of diving and hyperbaric chamber physicians	Gentner Verlag, book contribution: diving medicine	No	self-employed pediatric physician	Mandate holder of the DLRG<br>Employer: self-employed pediatric physician and diving medical examinations<br>Member: VDST	Diving and hyperbaric medicine<br>Minor restriction on function in the guideline group for level 1 (working group leadership)
Philipp Wolf	No	No	No	No	No	No	Mandate holder of the German Red Cross	No restriction on function in the guideline group

	Consultant or expert activity	Cooperation on a scientific advisory board	Paid lecturing or training activities	Paid authorship or coauthorship	Research projects/conducting clinical studies	Ownership interests (patent, copyright, share ownership)	Indirect interests	Guideline topics affected by COI<br>Classification in terms of relevance<br>Impact
							Employer: University of Regensburg	
Oliver Müller	No	No	Instructor for the training of diving and hyperbaric chamber physicians	No	No	No	Mandate holder of the DGAI<br>Employer: Vivantes Klinikum im Friedrichshain<br>Medical Director, Hyperbaric Oxygen Therapy and Diving Medicine Division<br>Member: GTÜM, DIVI, EUBS	Diving and hyperbaric medicine<br>Moderate restriction on in level 2 guideline group (abstention on HBOT recommendations)
Thorsten Janisch	No	No	Instructor for the training of diving and hyperbaric chamber physicians	Thieme Verlag, book contribution: hyperbaric medicine	No	No	Acting Mandate holder of the DGAI<br>Employer: B.A.D. Gesundheitsvorsorge und Sicherheitstechnik GmbH, physician in advanced training for occupational medicine<br>Member: GTÜM, EUBS, DIVI<br>Freelance physician, HBO Center Euregio Aachen	Diving and hyperbaric medicine<br>Minor restriction on function in the guideline group for level 1 (working group leadership)
Johannes Naser	No	No	Instructor for the training of diving and hyperbaric chamber physicians	No	No	No	Mandate holder of the BDA<br>Employer: Stauferklinikum, Mutlangen<br>Member: GTÜM<br>Freelance physician, Klinikum Ludwigsburg	Diving and hyperbaric medicine<br>Minor restriction on function in the guideline group for level 1 (working group leadership)
Susanne Blödt	No	No	No	No	No	No	Moderation of the guideline group (no voting rights)<br>Employer: AWMF	No restriction on function in the guideline group
Cathleen Muche-Borowski	No	No	No	No	No	No	Moderation of the guideline group (no voting rights)<br>Employer: University Hospital Hamburg-Eppendorf	No restriction on function in the guideline group

AWMF
